# Mass Spectrometry of Transferrin and Apolipoprotein CIII from Dried Blood Spots for Congenital Disorders of Glycosylation

**DOI:** 10.5702/massspectrometry.A0113

**Published:** 2022-12-28

**Authors:** Yoshinao Wada, Machiko Kadoya, Nobuhiko Okamoto

**Affiliations:** 1 Department of Obstetric Medicine, Osaka Women’s and Children’s Hospital (OWCH), 840 Murodo-cho, Izumi, Osaka 594–1101, Japan; 2 Department of Molecular Medicine, Osaka Women’s and Children’s Hospital (OWCH), 840 Murodo-cho, Izumi, Osaka 594–1101, Japan; 3 Department of Medical Genetics, Osaka Women’s and Children’s Hospital (OWCH), 840 Murodo-cho, Izumi, Osaka 594–1101, Japan

**Keywords:** congenital disorders of glycosylation, dried blood spot, transferrin, apolipoprotein CIII, glycoform

## Abstract

Dried blood spot (DBS) is the standard specimen for the newborn screening of inborn errors of metabolism (IEM) by tandem mass spectrometry. Availability of DBS for the mass spectrometric analysis of the diagnostic marker proteins, transferrin (Tf) and apolipoprotein CIII (apoCIII), of congenital disorders of glycosylation (CDG) was examined. Recovery of Tf from DBS was only slightly reduced compared with fresh serum. Although oxidation of the core polypeptides was observed, glycans of Tf and apoCIII were unaffected by storage of DBS in the ambient environment for at least 1 month. The combination of DBS and the triple quadrupole mass spectrometer used for IEM screening was sufficient to characterize the aberrant glycoprofiles of Tf and apoCIII in CDG. DBS or dried serum spot on filter paper can reduce the cost of sample transportation and potentially promote mass spectrometric screening of CDG.

## INTRODUCTION

Standard newborn screening (NBS) programs use dried blood spot (DBS) sampling for metabolite analysis.^1)^ DBS sampling is a simple, cost-effective and minimally invasive alternative to venipuncture, facilitates sample collection and reduces the burden of sample storage and shipping. DBS is also used for measurement of small pharmaceutical compounds for therapeutic drug monitoring,^2)^ and the application extends to the public health and epidemiological research, such as monitoring of environmental exposure biomarkers.^3)^ Furthermore, proteins such as immunoglobulins in DBS are measured by immunoassay: *e.g.* to monitor SARS-CoV-2 antibody prevalence in pregnant individuals by detecting maternal IgG antibodies in newborn DBS samples.^4)^ This suggests that proteins in DBS can be utilized for population or screening studies.

Congenital disorders of glycosylation (CDG) are an expanding group of genetic diseases affecting the synthesis of glycoconjugates (glycoprotein, glycosphingolipid or glycosylphosphatidylinositol anchor).^5)^ Diagnosis of CDG is a challenge due to the diverse and overlapping clinical signs and symptoms from multiple organ systems. Transferrin (Tf) and apolipoprotein CIII (apoCIII) are biomarkers of *N*- and *O*-glycosylation disorders, respectively, and an abnormality in the glycans of these glycoproteins supports the diagnosis of CDG. While isoelectric focusing (IEF) has been used to detect abnormal glycosylation of Tf since 1984,^6)^ mass spectrometry (MS) has demonstrated a distinct performance in glycoform analysis or glycoprofiling since its first application to CDG in 1992.^7,8)^ The major hurdle to introducing MS for a diagnostic or screening purpose is the instrument cost and operational skills. However, triple quadrupole-type mass spectrometers with an electrospray ionization source are currently used world-wide for the NBS of inborn errors of metabolism (IEM).^9,10)^ We have previously demonstrated that a quadrupole mass spectrometer, which was used for NBS and not equipped with a high resolution time-of-flight mass analyzer, can analyze Tf and apoCIII for CDG.^11,12)^

In recent reports, Tf in DBS was analyzed by IEF, and DBS was not inferior to serum samples for diagnosis and therapeutic monitoring of CDG.^13,14)^ In this study, the availability of DBS for the MS of Tf and apoCIII was investigated to promote the screening for *N*- and *O*-glycosylation disorders.

## MATERIALS AND METHODS

### Subjects and ethical approval

Blood or serum samples without personally identifiable information were delivered from the doctors in charge of the Osaka Women’s and Children’s Hospital (OWCH) for diagnosis of CDG. They were affected by developmental delay or multisystem disease of unknown etiology. This study has been approved by the institutional review board of OWCH. Patient sera stored at −80°C for 2 months to 3 years were used in this study.

### Preparation of DBS on filter paper

A fifty μL aliquot of blood or serum was spotted to form a 10 mm-diameter spot onto a Whatman 903 Guthrie paper, or No. 514A chromatography paper (0.32 mm thickness, TOYO Roshi, Tokyo, Japan) equivalent to Whatman 3MM. After drying, the filter papers were left on the laboratory bench for 3 days to 1 month before analysis. The room temperature was 20–22°C and humidity was 30–50%.

For old serum samples, reconstitution of a whole blood sample was performed as follows. Venous blood from a healthy volunteer was collected in an EDTA-treated tube. The cellular and plasma layers were separated by centrifugation for 10 min at 2,000×g. The packed cell was prepared by centrifugation after being washed twice with phosphate-buffered saline (PBS). Whole blood was reconstituted by mixing the stored serum and packed cell layer in a 3 : 2 volume ratio and spotted onto filter paper.

### Immunopurification of Tf and apoCIII from DBS

One whole blood spot approximately 10 mm in diameter was excised, cut into 4 pieces and placed in an Eppendorf tube. One mL of PBS was added to the tube and incubated for 1 h at room temperature with gentle agitation. The solution was transferred to another tube. After centrifugation for 5 min at 20,000×g, the solution was passed through a 0.22 μm filter to remove paper debris and subjected to immunopurification as described previously.^11,12)^ Briefly, an affinity gel coupled with polyclonal antibody to human Tf (DAKO, Glostrup, Denmark) or human apoCIII (Academy Bio-Medical Co., Houston, TX, USA) was prepared using NHS-activated Sepharose (HiTrap HP, GE Healthcare, Piscataway, NJ, USA). A ten μL portion of serum was mixed with a 20-μL slurry of the antibody-coupled Sepharose in 0.5 mL of PBS, and the solution was incubated at 4°C for 30 min. After washing with PBS, Tf or apoCIII was eluted from the Sepharose with 0.1 M glycine–HCl buffer at pH 2.5. Protein concentration was calculated by the Bradford method.

### Mass spectrometry

Liquid chromatography MS was carried out by an API4500 ESI-triple quadrupole mass spectrometer (Sciex, Framingham, MA, USA), which was previously used for the IEM screening and not equipped with a time-of-flight mass analyzer, connected to a C4 reversed phase column (2 mm diameter and 10 mm length, GL Sciences, Tokyo, Japan). After sample injection, the column was washed with 0.1% formic acid at a flow rate of 0.2 mL/min, and then eluted with 60% acetonitrile/0.1% formic acid at a flow rate of 0.05 mL/min. For apoCIII, a short gradient of increasing acetonitrile was applied to avoid co-elution of contaminating albumin and apolipoprotein CII. API4500 was operated in the positive Q1 MS mode with the optimized parameters as follows. For Tf, gas temperature was at 150°C, curtain gas pressure was 10 psi, ion source gas pressure was 16 psi, IonSpray voltage was 5.5 kV, declustering potential was 150 V, and entrance potential was 10 V. The full scan range was set from 1780 to 2000, and the scan rate was 10 Da/s. For apoCIII, gas temperature was at 120°C, curtain gas pressure was 10 psi, ion source gas pressure was 16 psi, IonSpray voltage was 5.5 kV, declustering potential was 100 V, and entrance potential was 10 V. The full scan range was set from 790 to 1650, and scan rate was 200 Da/s. Mass calibration was conducted using polypropylene glycol. The zero-charge mass spectrum was generated by the Promass protein deconvolution software (Thermo-Fisher Scientific, Waltham, MA, USA), by calculating mass spectral data in the *m*/*z* 1780–2000 and *m*/*z* 973–1650 ranges for Tf (40–44 charged ions) and apoCIII (6–9 charged ions), respectively.

## RESULTS AND DISCUSSION

### Recovery of Tf from DBS

Recovery of Tf and apoCIII from DBS as well as their stability during storage in DBS are a critical issue for reliable and effective analysis. In a report on 34 NBS markers, the humidity was the key parameter of most compounds to be stable at 37°C.^15)^ The metabolites such as amino acids, galactose and carnitines were stable for one month under low humidity. Regarding proteins, enzymatic activity of galactose-1-phosphate uridyl-transferase and biotinidase was lost >60% of their initial levels in DBS for 32 days at 37°C even in the low humidity environment. Immunoreactivity levels of the 27 kDa protein trypsinogen, a marker of cystic fibrosis, decreased by 18% during storage.^15)^ This indicates that protein degradation or denaturation occurs to some extent in DBS. Also, the recovery rate of proteins from DBS samples should be concerned, even after short-term storage.

First, the amount of Tf (80 kDa) recovered from DBS was measured. As shown in Table 1, recoveries from DBS or dried serum spot on filter paper were slightly reduced compared to those from fresh serum to 6% and 20%, respectively.

**Table 1:** Table 1. Recovery from dried blood sample.

50 μL each	Protein (wt, μg)
Serum (wet)	48.6
Dried serum (No 514A paper)	45.4
Dried serum (Guthrie paper)	45.7
DBS (No 514A paper)	39.1
DBS (Guthrie paper)	36.0

DBS: dried blood spot (whole blood)

### MS of Tf and apoCIII from DBS

In the recent reports, the IEF profile of Tf from DBS was almost the same as that from serum.^13,14)^ This indicates that the charge state of the Tf glycans is unchanged, but does not necessarily guarantee that the glycan structure is intact. To address the issue, the glycoform and glycoprofile of Tf were evaluated by MS. In this study, dried serum spot on filter paper was also analyzed because serum is currently used for the CDG testing.

**Figure 1:**
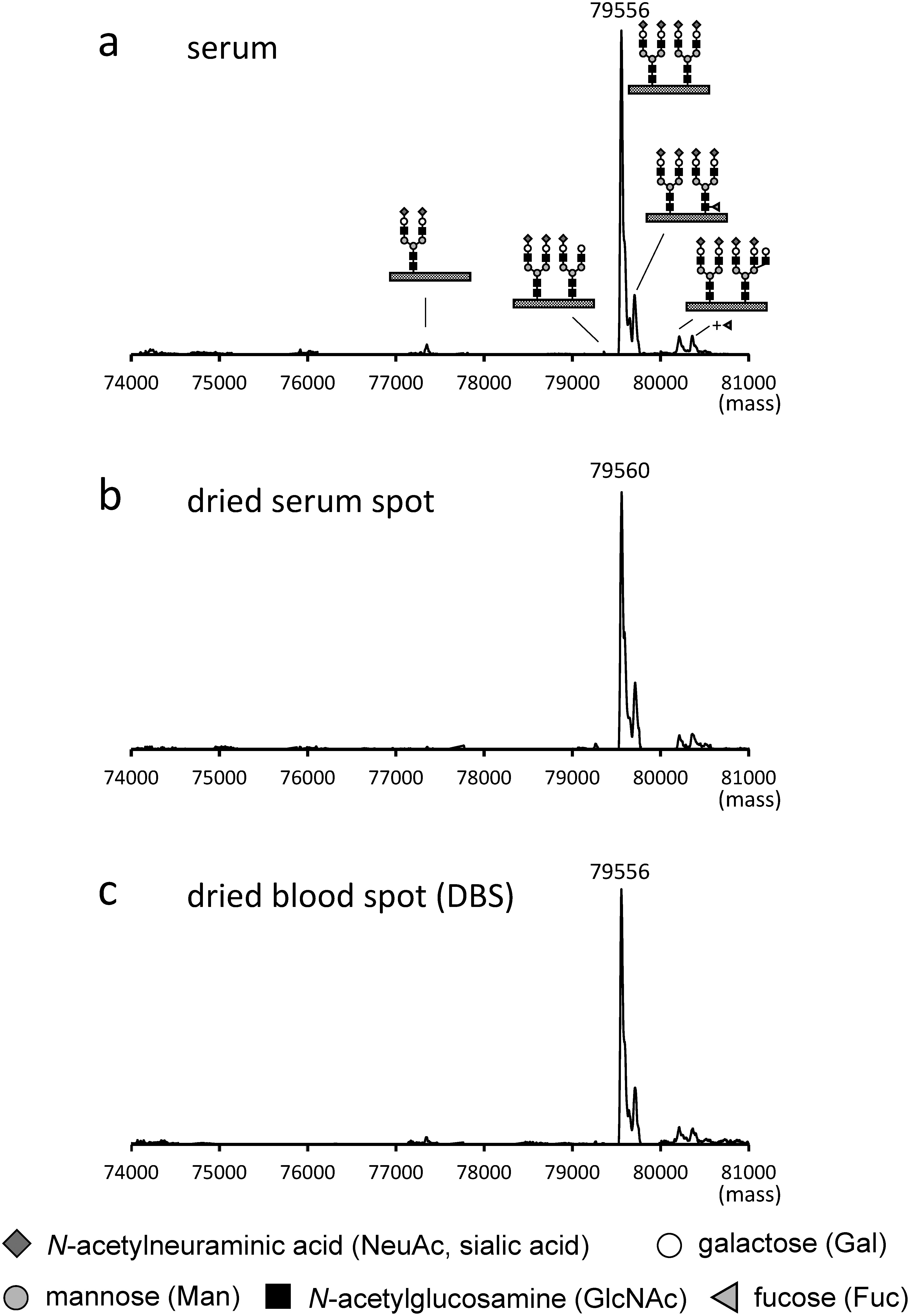
Fig. 1. Charge-deconvoluted spectra of Tf from various samples.

In Fig. 1a, the Tf molecule with two biantennary complex-type glycans was the predominant species, and those with fucosylated and triantennary glycans were observed. Additionally, Tf molecules lacking sialic acid or one of the glycans were identified as very small peaks. The mass spectra from dried serum spot and DBS were the same as fresh serum (Figs. 1b and 1c).

a. fresh serum, b. dried serum spot on filter paper, c. dried blood spot on filter paper. The spectrum data files are available in J-STAGE Data. https://doi.org/10.50893/data.massspectrometry.21739334.

**Figure 2:**
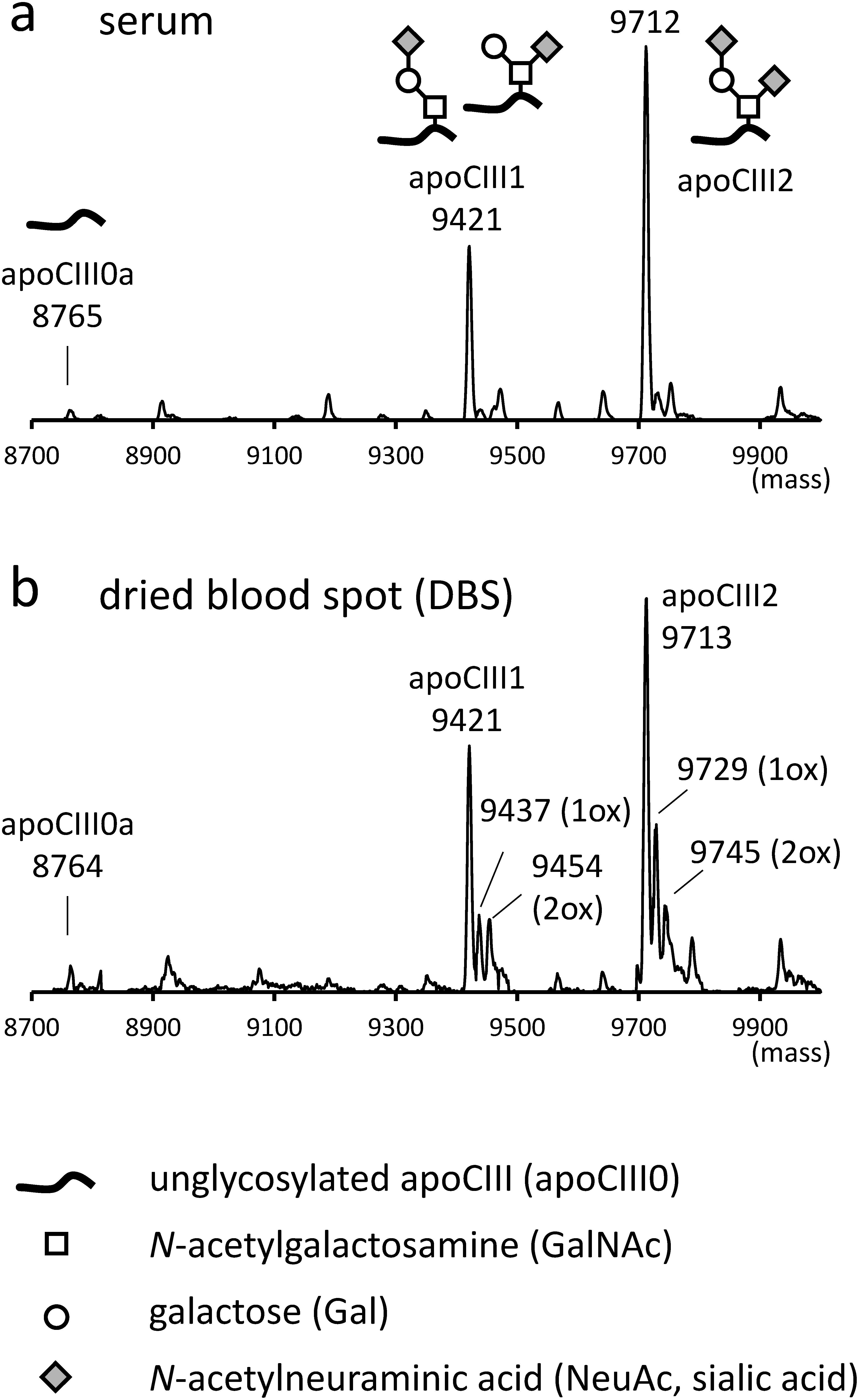
Fig. 2. Charge-deconvoluted spectra of apoCIII from fresh wet serum or DBS.

ApoCIII is a mucin-type *O*-glycosylation marker of CDG.^16,17)^ ApoCIII0a, apoCIII1 and apoCIII2 are key diagnostic molecules whose relative abundances are important diagnostic parameters^11)^ (Fig. 2a). DBS yielded nearly identical profiles for these apoCIII molecules, while oxidation at methionine residues was observed in the DBS sample^18)^ (Fig. 2b). ApoCIII from dried serum spot samples presented the same profile as DBS (data not shown). The oxidized molecules were not identified in the mass spectra of Tf from DBS or dried serum spot (Figs. 1b and 1c), probably due to an insufficient resolving power for large molecules. However, masses of Tf measured from DBS or dried serum spot samples suggested that the unoxidized molecule was the predominant species. These findings indicated that the dried samples can be stored in the ambient environment for at least one month without significantly affecting the MS of Tf and apoCIII.

a. serum, b. DBS. ox, the number of gained oxygen by oxidation of methionine residues. The spectrum data files are available in J-STAGE Data. https://doi.org/10.50893/data.massspectrometry.21739382.

### Glycoprofiling from DBS samples

In this study, fresh whole blood samples were not available from CDG patients. Alternatively, DBS from CDG patients were prepared by mixing patient sera with blood cells (mainly red blood cells) from a control subject and spotted on filter paper. The mass spectra of Tf and apoCIII from reconstituted DBS were the same as those from dried serum spot (data not shown).

**Figure 3:**
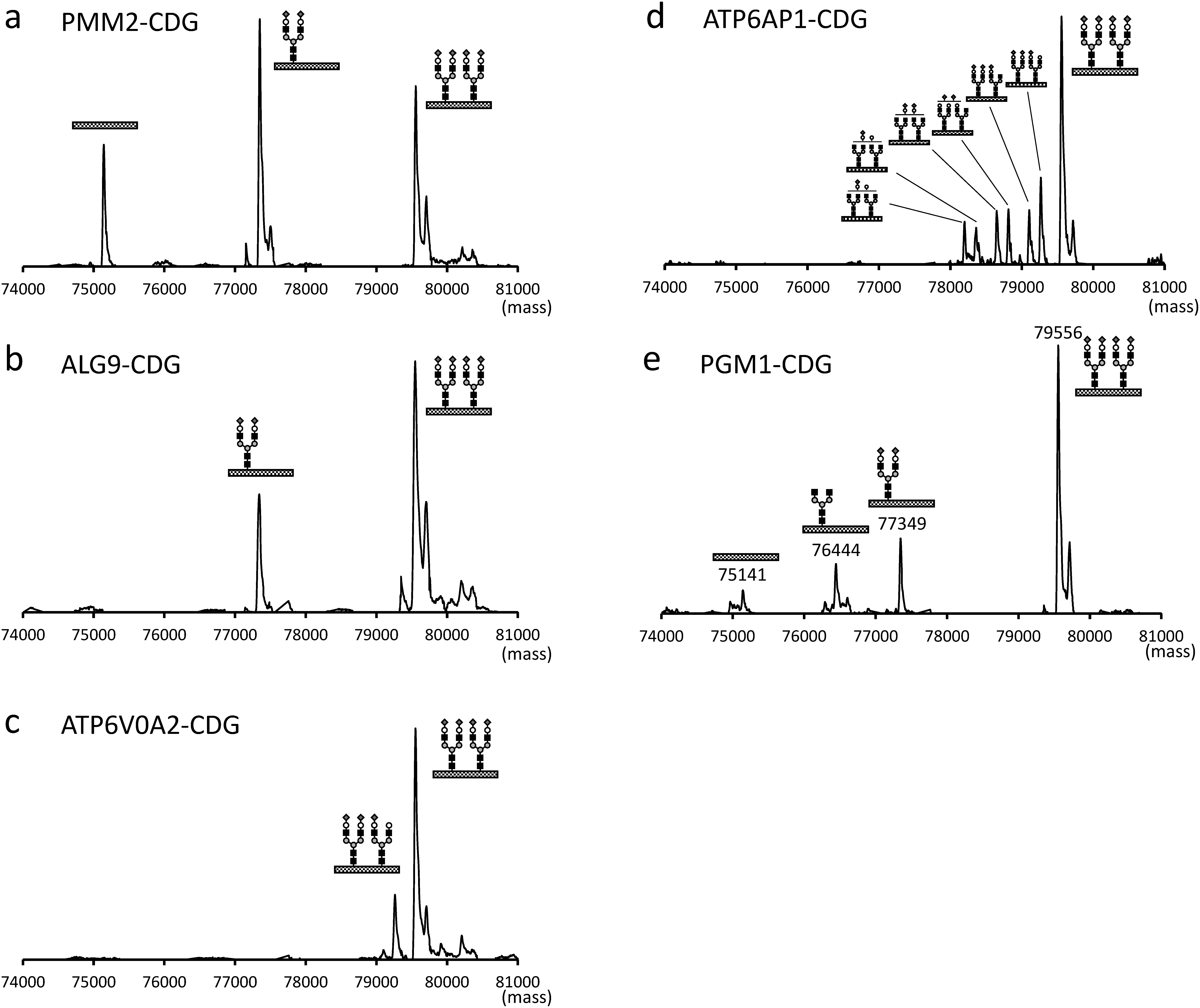
Fig. 3. Charge-deconvoluted Tf spectra of various types of CDG.

**Figure 4:**
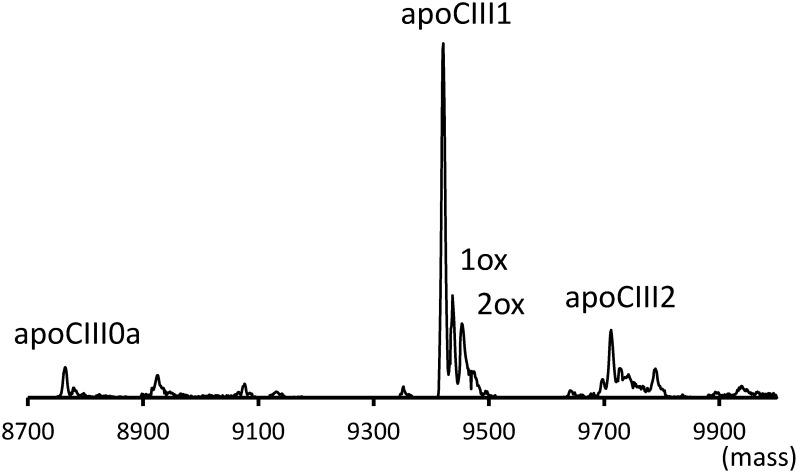
Fig. 4. Charge-deconvoluted apoCIII spectra of ATP6V0A2-CDG.

To demonstrate that DBS sampling is viable for finding CDG, Tf and apoCIII from DBS or dried serum spot of CDG patients were analyzed by MS (Fig. 3). The presence of Tf molecules lacking one or two glycans indicates an early *N*-glycosylation pathway defect in the endoplasmic reticulum. These abnormal Tf molecules were observed or increased in PMM2-CDG (OMIM #601785), the most common type of CDG, and ALG9-CDG (OMIM #608776) (Figs. 3a and 3b). In the late glycosylation pathway, *N*-glycans are processed in the Golgi apparatus, where the deficiency of glycoenzymes and defects of the Golgi integrity and the vesicular dynamics such as membrane fusion and trafficking impair glycan processing and thus accumulate immature glycans.^19,20)^ ATP6V0A2 (OMIM #219200) and ATP6AP1-CDG (OMIM #300972) belong to this group, and the responsible genes encode the components of the vacuolar ATPase protein pump which is involved in luminal acidification of secretory vesicles and membrane trafficking. Undersialylation was a typical abnormality in these cases (Figs. 3c and 3d), and galactosylation was also impaired in ATP6AP1-CDG. In PGM1-CDG (OMIM #614921), depletion of sugar nucleotides, UDP-glucose and UDP-galactose, impairs the early and late *N*-glycosylation pathways, respectively.^21)^ The mass spectrum identified the unique profile of Tf from this type of CDG in Fig. 1e. Finally, an example of abnormal apoCIII glycoprofile was provided by ATP6V0A2-CDG, which is associated with *O*- as well as *N*-glycosylation defects. In Fig. 4, reduced levels of the apoCIII2 isoform indicated undersialylation of mucin-type *O*-glycans.

a. PMM2-CDG, Tf (rDBS), b. ALG9-CDG, Tf (DSS), c. ATP6V0A2-CDG, Tf (rDBS), d. ATP6AP1-CDG, Tf (DSS), e. PGM1-CDG, Tf (DSS). rDBS, reconstituted dried blood spot. DSS, dried serum spot. The spectrum data files are available in J-STAGE Data. https://doi.org/10.50893/data.massspectrometry.21739391.

The sample was reconstituted dried blood spot. The spectrum data files are available in J-STAGE Data. https://doi.org/10.50893/data.massspectrometry.21739406.

## CONCLUSION

Glycoforms of Tf and apoCIII recovered from DBS or dried serum spots were apparently intact during storage for at least 1 month. MS of patient DBS samples characterized aberrant *N*- and *O*-glycosylation in different types of CDG. The simple sampling and facile transport of DBS coupled with the standard mass spectrometer used in current NBS programs may help CDG detection and facilitate screening for glycosylation disorders.

## Data Availability

The spectrum data files are available in J-STAGE Data.

## Notes

Mass Spectrom (Tokyo) 2022; 11(1): A0113
